# School health implementation tools: a mixed methods evaluation of factors influencing their use

**DOI:** 10.1186/s13012-018-0738-5

**Published:** 2018-03-20

**Authors:** Jennifer Leeman, Jean L. Wiecha, Maihan Vu, Jonathan L. Blitstein, Sallie Allgood, Sarah Lee, Caitlin Merlo

**Affiliations:** 1 School of Nursing, University of North Carolina at Chapel Hill, Chapel Hill, USA; 2 RTI International, Durham, USA; 3 Center for Health Promotion & Disease Prevention, University of North Carolina at Chapel Hill, Chapel Hill, USA; 4 Division of Population Health, School Health Branch, Centers for Disease Control and Prevention, Atlanta, USA

**Keywords:** Implementation tools, School health, Consolidated framework for implementation research, Interactive systems framework

## Abstract

**Background:**

The U.S. Centers for Disease Control and Prevention (CDC) develops tools to support implementation of evidence-based interventions for school health. To advance understanding of factors influencing the use of these implementation tools, we conducted an evaluation of state, school district, and local school staffs’ use of four CDC tools to support implementation of physical activity, nutrition, health education, and parent engagement. Two frameworks guided the evaluation: Interactive Systems Framework (ISF) for Dissemination and Implementation and Consolidated Framework for Implementation Research (CFIR).

**Methods:**

The evaluation applied a mixed methods, cross-sectional design that included online surveys (*n* = 69 state staff from 43 states), phone interviews (*n* = 13 state staff from 6 states), and in-person interviews (*n* = 90 district and school staff from 8 districts in 5 states). Descriptive analyses were applied to surveys and content analysis to interviews.

**Results:**

The survey found that the majority of state staff surveyed was aware of three of the CDC tools but most were knowledgeable and confident in their ability to use only two. These same two tools were the ones for which states were most likely to have provided training and technical assistance in the past year. Interviews provided insight into how tools were used and why use varied, with themes organized within the ISF domain “support strategies” (e.g., training, technical assistance) and four CFIR domains: (1) characteristics of tools, (2) inner setting, (3) outer setting, and (4) individuals. Overall, tools were valued for the credibility of their source (CDC) and evidence strength and quality. Respondents reported that tools were too complex for use by school staff. However, if tools were adaptable and compatible with inner and outer setting factors, state and district staff were willing and able to adapt tools for school use.

**Conclusions:**

Implementation tools are essential to supporting broad-scale implementation of evidence-based interventions. This study illustrates how CFIR and ISF might be applied to evaluate factors influencing tools’ use and provides recommendations for designing tools to fit within the multi-tiered systems involved in promoting, supporting, and implementing evidence-based interventions in schools. Findings have relevance for the design of implementation tools for use by other multi-tiered systems.

## Background

By virtue of their reach and mission, schools can have a holistic influence on children and youth, fostering intellectual as well as social, emotional, and physical development. Over 50 million students attend over 98,300 public k-12 schools annually for 180 days per year for an average of 6.6 h per day [1, 2]. The unparalleled reach schools have offers great efficiency in creating health-promoting environments and programs. And, over time, an agreement has emerged among educators and public health professionals endorsing the importance of the role of schools in promoting healthy behaviors [3]. Schools can promote healthy behaviors through health education and by providing access to healthy foods and adequate, age-appropriate physical activity [4].

Federal agencies, including the U.S. Department of Agriculture and Centers for Disease Control and Prevention, have developed a number of “implementation tools” to promote and support the integration of evidence-based interventions (EBIs) into US schools, with EBIs defined broadly to include programs, practices, policies, and guidelines [5]. “Implementation tools” include electronic and print resources that summarize and organize information about EBIs and provide guidance on how to select, adapt, implement, and evaluate those EBIs in practice [6–8]. Despite the prevalence of implementation tools, little is known about how they are used or factors that may influence their use in non-clinical settings [8].

To better understand how implementation tools are used in schools, we conducted an evaluation of how staff working in state departments of health and education, school district offices, and local schools are using the following CDC implementation tools:

1. *Comprehensive School Physical Activity Program (CSPAP) Guide*. Released in January 2014, CSPAP provides guidance for schools and school districts to develop, implement, and evaluate EBIs to increase physical activity before, during, and after school and to equip students for a lifetime of physical activity [9].
2. *School Health Guidelines to Promote Healthy Eating and Physical Activity (SHG).* Released in September 2011, SHG is a synthesis of research findings and best practices into nine guidelines for promoting healthy eating and physical activity in schools [10].
3. *Health Education Curriculum Analysis Tool (HECAT)*. The most recent version of HECAT was released in 2012 and provides guidance, appraisal tools, and resources to select, develop, or improve health education curricula [11].
4. *Parents for Healthy Schools (P4HS)*. Released in November 2015, P4HS provides tools schools can use to engage parents and school groups that work with parents (e.g., school health councils, Parent Teacher Associations) in efforts related to school nutrition, physical activity, and management of chronic conditions [12].

### Evaluation framework

**Fig. 1:**
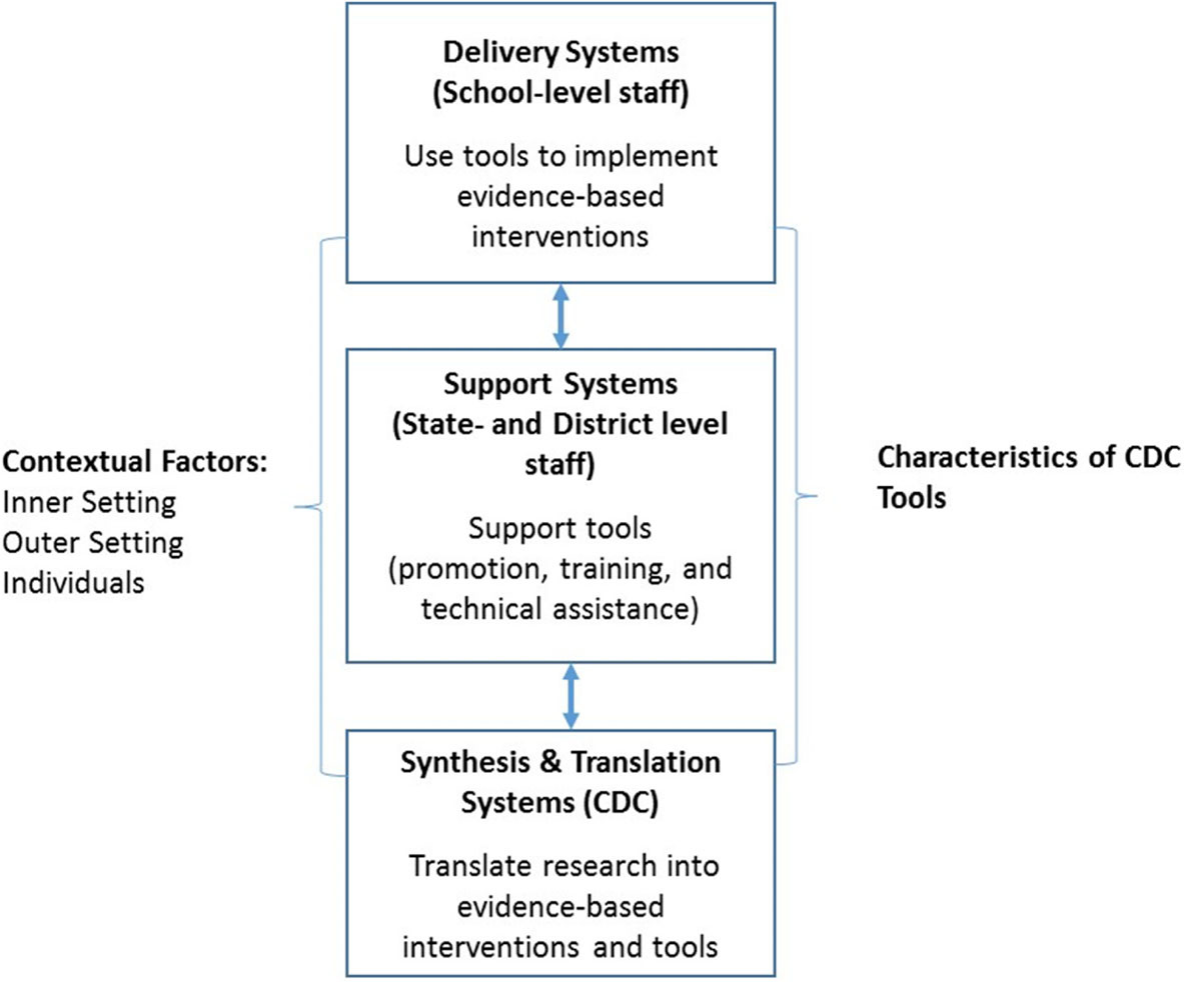
Evaluation Framework [6, 13, 14]

The framework guiding this evaluation (Fig. 1) integrates the Interactive Systems Framework (ISF) for Dissemination and Implementation [13] and the Consolidated Framework for Implementation Research (CFIR) [14]. The ISF describes three systems that interact to translate research findings into practice: delivery systems, synthesis and translation systems, and support systems. As applied to this study, schools are the *delivery system*, and children and their parents are the primary recipients of the services delivered. The CDC functions as a *synthesis and translation system* that synthesizes research findings and translates them into EBIs and implementation tools to improve school health. Staff in state departments of public health and education and school districts function as *support systems* that promote the implementation tools and provide training and technical assistance to support their use [13]. The ISF was applied to differentiate between two types of implementation tool users—those working in support systems versus those working in delivery systems. The CFIR was applied to categorize factors that may facilitate or hinder use of implementation of tools within the following four domains:

- *Characteristics of the implementation tool*. Although in CFIR, this domain applies to interventions, for this study, we applied it to characteristics of implementation tools. Examples of this category’s constructs include “evidence strength and quality,” “adaptability,” and “complexity.”
- *Outer setting*. This domain includes an organization or system’s political and social context. Examples of relevant constructs include “external policy” and “cosmopolitanism” (i.e., the extent to which organizations are networked with other organizations.)
- *Inner setting*. This domain includes the structural, political, and cultural context within an organization. Examples of relevant constructs include “leadership engagement” and “available resources.”
- *Characteristics of individuals*. In this study, “individuals” include those providing support for the tools (i.e., state and district staff) as well as those using the tools to implement EBIs (school staff). Examples of constructs include “knowledge” and “self-efficacy.”

We replaced CFIR’s fifth domain, “processes”, with the ISF’s concept of “innovation support” strategies because it better captured the study’s focus on external, in addition to, internal implementation processes [6]. We defined support strategies to include the strategies an external organization uses to improve a delivery system’s implementation of either EBIs or implementation tools (e.g., promotion, training, and technical assistance) [6, 15].

The evaluation addressed the following questions:

1. What types of promotion, training, and technical assistance are support system staff using?
2. For what purposes are support and delivery system staff using the CDC’s tools?
3. What contextual factors influence support and delivery system staffs’ use of the CDC’s tools?
4. What characteristics of the CDC’s tools influence their use by support and delivery system staff?

## Methods

### Design

The evaluation applied a mixed methods, cross-sectional design to collect data from staff in state departments of health and education (online survey and phone interviews) and in school districts and local schools (in-person interviews and focus groups). A stakeholder advisory board, consisting of academic researchers, CDC staff, and state coordinators of CDC’s school health branch-funded programs, provided input into all phases of the evaluation. The study was reviewed by the University of North Carolina at Chapel Hill (UNC-CH) and RTI International Institutional Review Boards and determined to be exempt and also was reviewed and approved by the U.S. Office of Management and Budget.

### Sample

#### State staff survey

The CDC provided contact information for all 178 state staff coordinating CDC-funded school health programs. Sixty-nine staff responded to the online survey (38.8% response rate), representing 43 states, which included the District of Columbia. Table 1 provides an overview of the survey participants, the majority of whom had a master’s degree or higher (*n* = 48, 69.6%) and had been in their current position for at least 2 years (72.4%) and at their agency for at least 5 years (56.5%). Forty-two (60.8%) respondents worked in departments of health and 27 (29.2%) in departments of education.

**Table 1:** Respondent characteristics, CDC School Health Tools Survey, 2016

Characteristic	Total (*N* = 69)		Department of Health (*N* = 42)		Department of Education (*N* = 27)	
	*n*	%	*n*	%	*n*	%
Highest degree attained						
Bachelor’s level	20	29.0	15	35.7	5	18.5
Master’s level	41	59.4	25	59.5	16	59.3
Advanced degree	7	10.1	2	4.8	5	18.5
Not reported	1	1.4	0	0.0	1	3.7
How long in current position						
Less than 1 year	9	13.0	7	16.7	2	7.4
More than 1 year, less than 2 years	10	14.5	6	14.3	4	14.8
More than 2 years, less than 5 years	20	29.0	14	33.3	6	22.2
More than 5 years, less than 10 years	15	21.7	9	21.4	6	22.2
10 years or more	15	21.7	6	14.3	9	33.3
How long in current agency						
Less than 1 year	3	4.3	1	2.4	2	7.4
More than 1 year, less than 2 years	7	10.1	4	9.5	3	11.1
More than 2 years, less than 5 years	20	29.0	14	33.3	6	22.2
More than 5 years, less than 10 years	17	24.6	11	26.2	6	22.2
10 year or more	22	31.9	12	28.6	10	37.0
How long in school health field						
Less than 1 year	11	15.9	8	19.0	3	11.1
More than 1 year, less than 2 years	4	5.8	3	7.1	1	3.7
More than 2 years, less than 5 years	10	14.5	9	21.4	1	3.7
More than 5 years, less than 10 years	15	21.7	13	31.0	2	7.4
10 years or more	29	42.0	9	21.4	20	74.1

#### State staff interviews

The research team phone interviewed a subset of 13 staff from six states that were purposefully selected based on online survey findings indicating high performance (i.e., promoting at least three of the four CDC tools) and geographic representativeness (states in western, midwestern, southern, and northern USA). All invited state staff participated in the interviews (100% response rate). Thirteen state staff participated in phone interviews, including 9 (69.2%) in departments of health and 4 (30.7%) in departments of education.

#### District interviews

We use the term “district” to refer broadly to any regional entity within a state’s school system. State staff who participated in phone interviews were asked to identify one or two districts in their state that exemplified improvements to school health and, where possible, to identify districts in both rural and urban areas. State staff then provided the name of a contact person for each district who identified three or more district and local school staff who were actively involved in school health. Staff in five of the six states identified eight districts (one state had no districts available to participate). In the remaining five states, all invited districts agreed to participate. The districts identified 90 participants; 27 of whom participated in in-person interviews and 63 in focus groups. Forty (44.4%) worked in district offices, predominantly in the roles of health services directors, nutritionists, and health and wellness coordinators, and 50 (55.6%) worked in local schools as principals, assistant principals, health and physical educators, and classroom teachers.

### Measures

#### Survey

The online survey covered three main areas: (1) descriptive information about respondents and their employing agency, (2) respondents’ awareness and perceptions of each tool, and (3) state provision of support (e.g., training) for each tool. Survey items were adapted from field-tested instruments developed by the CDC and UNC [16] and included dichotomous (yes/no), multiple response, ordinal rating scale, and open-ended questions. New items also were developed to reflect the evaluation framework. Survey questions were assessed in cognitive interviews with three staff with experience coordinating their state’s school health programs. Surveys were then refined to ensure that items were easy to understand, and response options were clear, exhaustive, and mutually exclusive [17]. The survey was programmed in SurveyGizmo and administered in a web-based, online format that included a core module and four tool-specific modules that respondents completed or skipped according to whether they reported awareness of the tool. The survey was distributed via an email invitation followed by two reminder emails and was open from October 12, 2016, to December 10, 2016.

#### Interview guides

Semi-structured interview guides were created and pilot tested with three state CDC school health program coordinators. The interview guide for state staff consisted of open-ended questions that asked which tools states were using, perceptions of the tools, partnerships formed to promote and support the tool, how districts and schools were using the tool, and challenges encountered. The guide for school district and local school staff covered the same topics but was designed to gain more in-depth information about participants’ experience using the one or two tools they identified as most familiar and/or frequently used. In-person interviews were conducted between December 2016 and April 2017.

### Analysis

#### Survey

Data analysis consisted of descriptive statistics including frequencies, proportions, means, and standard deviations. Data are reported by respondent (*n* = 69) and by state (*n* = 43). For states with multiple respondents, we used state data from the respondent who reported the highest number of job responsibilities (e.g., promotion, training) and highest self-reported level of influence on agency decisions related to school health.

#### Interviews

In-person interviews were recorded and transcribed. A directed form of content analysis was used to analyze data, guided by the project’s evaluation framework [18]. The CFIR was applied to develop an initial list of codes related to contextual factors and characteristics of the CDC tools [14]. During the coding process, inductively derived codes were developed as needed to fully capture all relevant information. The data were coded by two independent coders using the qualitative software management program ATLAS.ti. Coders met to compare and reconcile coding. Once coding was complete, data were put into a matrix and themes were identified across tools, states, and systems (support versus delivery).

## Results

Survey and interview findings are integrated and are organized by the study’s evaluation framework and questions. Unless identified as a survey finding, the findings reported were derived from the interviews.

### What types of promotion, training, and technical assistance are support systems using?

The provision of support strategies was multi-tiered, with state staff providing support to districts and both state and district staff providing support to schools and teachers. Support included three broad categories of strategies: promotion, training, and technical assistance.

#### Promotion

**Fig. 2:**
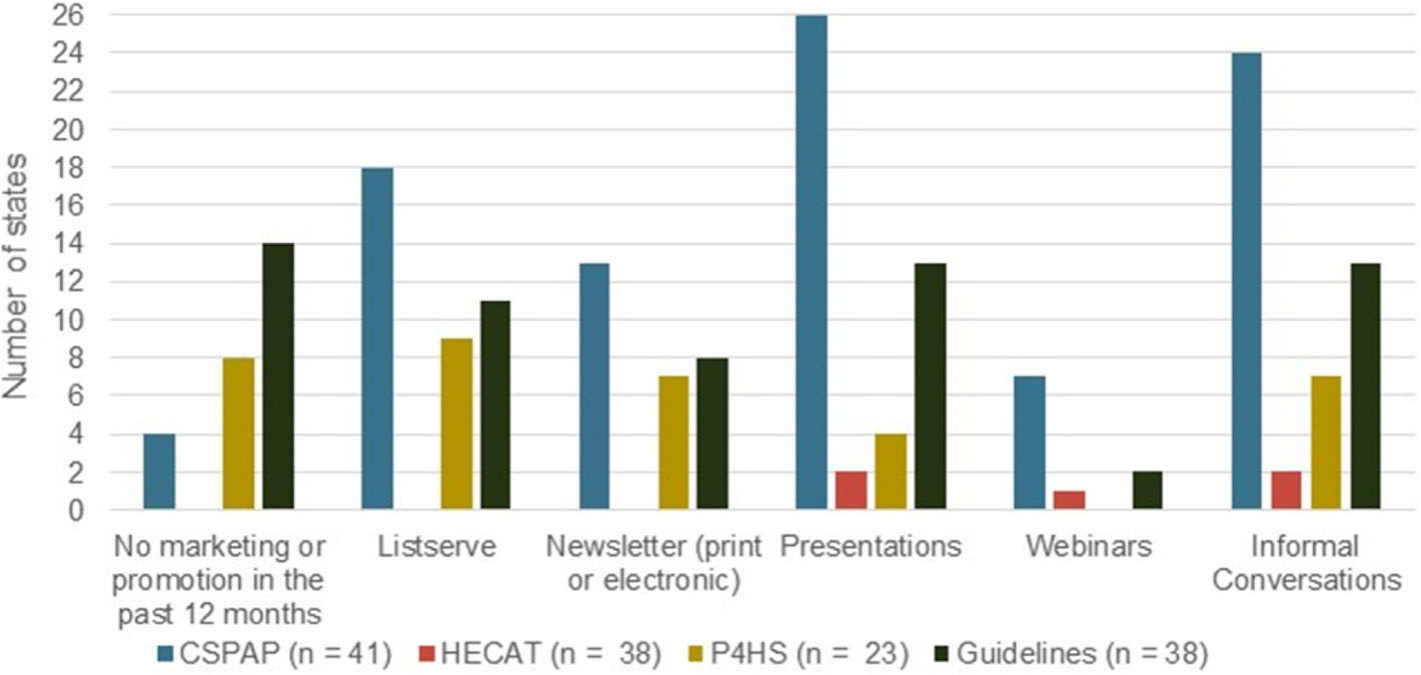
State data: methods used to promote awareness of tools in past 12 months, CDC School Health Tools Survey, 2016. Respondents indicated all methods applicable to each tool (i.e., check all that apply). CSPAP = Comprehensive School Physical Activity Program; HECAT = Health Education Curriculum Analysis Tool; P4HS = Parents for Healthy Schools; Guidelines = School Health Guidelines

Online survey findings show that the majority of states had promoted the CSPAP (*n* = 36; 83.7%) and SHG (*n* = 26; 60.5%) in the past 12 months with only a minority having promoted the HECAT (*n* = 15; 34.9%) or the P4HS (*n* = 15; 34.9%) (Table 2). States used a variety of strategies to promote the CDC’s tools. The most commonly used strategies were presentations, informal conversations, listservs, and newsletters (Fig. 2). When interviewed, state staff also reported providing links to the tools on their websites.

**Table 2:** State-level data: CDC School Health Tools Online Survey, 2016 (*N* = 43)

In the last 12 months, has your state engaged in…	CSPAP		HECAT		P4HS		SHG	
	*n*	%	*n*	%	*n*	%	*n*	%
… marketing/communications related to:	36	83.7	15	34.9	15	34.9	26	60.5
... training and/or technical assistance related to:	34	79.1	11	25.6	8	18.6	17	39.5

*CSPAP* Comprehensive School Physical Activity Program, *HECAT* Health Education Curriculum Analysis Tool, *P4HS* Parents for Healthy Schools, *SHG* School Health Guidelines

In interviews, state staff also reported incorporating CDC tools into their guidance documents and trainings and sharing tools for key partners to promote, for example, SHAPE America or the State Health and Physical Education Association.

#### Training

Over the past 12 months, the majority of states responding to the survey had provided training and/or technical assistance on the CSPAP (*n* = 34; 79.1%) and less than half on the SHG (*n* = 17; 39.5%), the HECAT (*n* = 11; 25.6%), and the P4HS (*n* = 8; 18.6%) (Table 2). In phone interviews, state staff reported that they often provided trainings in collaboration with partners including Playworks, SHAPE America, Alliance for a Healthier Generation, Action for Healthy Kids, and the Cooperative Extension. District staff also provided trainings for school staff and leveraged varying funding sources to send staff to trainings and to bring outside experts into schools to provide trainings on Smarter Lunchrooms [19], culinary skills, active classrooms, and active recess. State and district staff used a range of strategies to incentivize participation in trainings such as offering continuing education credits and requiring training as a condition of funding.

#### Technical assistance

State and district staff provided technical assistance by phone (one-to-one and conference calls) and in-person site visits. Five sub-themes emerged related to specific approaches used to provide technical assistance. They engaged staff in the local schools, coordinated support, adapted tools, assessed and provided feedback on school performance, and set achievable goals.

##### Engaged staff in the local schools

Developing relationships with those working in schools was key to getting them to use components of the CDC tools. In the words of one district wellness coordinator, “[It is] essential to build a relationship with that school…it’s the relationship piece. Your content doesn’t matter if you can’t get in the door.” School wellness champions were among the most important people to engage. Wellness champions included elementary school teachers, physical education teachers, and health education teachers who volunteered to advocate for health-supporting environments, policies, and practices within their schools. In the words of one participant, “Generally there's one person behind the scenes or maybe up front that's helping to move it, so that champion piece is pretty important.”

##### Coordinated external support

Numerous national, state, and regional organizations are supporting schools’ efforts to implement school health EBIs. State and district staff played a central role in coordinating and vetting these organizations and the support they provided. One state staff person spoke of her role as follows, “I have to filter a lot of this, because you just can’t throw all of this at school districts and expect them to digest it all with everything else that they have on their plates.”

Coordination also included leveraging funding from multiple sources to support school health including federal, state, and foundation funding among others. In some cases, this resulted in synergies that strengthened the school health work. In other cases, it resulted in staff being pulled in multiple different directions.

##### Adapted tools

State and district staff adapted tools to meet schools’ needs. Rather than providing schools with the full version of a CDC tool, they selected and extracted the information and guidance they viewed as best fitting a school’s needs and then, if needed, reformatted it to be user friendly. As one staff person noted, “When we get awesome information from CDC or NIH or WHO or whatever it is, we really gotta break it down. I mean we gotta water the crap [sic] out of it to send it and make it something that’s gonna mean anything to the people that we send it to.”

##### Assessed performance and provided feedback

Three of the six states that participated in interviews provided technical assistance in conjunction with formal assessments of school performance. These states asked schools to complete a self-review process and then integrated CDC tools into the technical assistance they provided to aid schools in addressing identified deficits or opportunities for improvement in their performance.

##### Set achievable goals

State and district staff advocated an approach to technical assistance that affirmed the work schools were already doing and then set small goals for improvement. In the words of one staff person,

I think sometimes when we come into the schools and we wanna do a program, we’re saying that they’re missing something. If we can approach them and say you’re doing an awesome job, and you’re doing X, Y and Z, but how about we just take it up a notch.

### For what purposes are support and delivery system staff using the CDC’s tools?

#### Support system use of tools

In phone and in-person interviews, state and district staff reported using the CDC tools for three primary purposes. These included (1) making the case for *why* school health EBIs should be adopted and (2) providing expert advice on *what EBIs* to adopt, in addition to (3) providing guidance on *how to* implement EBIs. As one district participant noted:

When the physical activity guidelines and all that came out… What it did is, it allowed us to continue moving forward our district policy, wellness policies, but also, it gave it more credibility, because the CDC was saying ‘Look, here it is. Now, here’s how you go about doing it.’

When used to provide “how to” guidance for implementing EBIs in schools, the tools were drawn on, for example, to guide schools in creating an action plan or communicating with parents.

#### Delivery system use of tools

The majority of local school staff were unfamiliar with the tools. Although they used components of the tools, they only did so after the support system staff extracted and translated components of the tools for their use. As a result, they were not familiar with the tools themselves. Staff in local schools went to a variety of other sources in search of ready-to-use tools, including Twitter, Facebook, Pinterest, YouTube, and other schools’ websites.

### What contextual factors influenced support system staffs’ use of the CDC’s tools?

Table 3 provides an overview of findings on factors that influenced the use of CDC’s tools with exemplar quotations from the interview data. State and district staffs’ use of tools was influenced by factors in CFIR’s Outer Setting and Characteristics of Individuals domains.

**Table 3:** Factors that influenced tool use, organized by ISF level and CFIR domains [14]

CFIR construct	Themes	Exemplar quotations
Support system		
Outer setting		
Cosmopolitanism	Interactions and partnerships with other organizations working to improve school health	“I will say that’s probably our strongest asset we have in [state] is that our partners in all of those different groups, we know and we work and collaborate and communicate on a pretty regular basis.”
External policy	• Shift from No Child Left Behind to Every Student Succeeds Act • CDC funding requirements and alignment with other federal agencies • State standards/regulation	“The areas that have caused us setbacks would be unintended consequences of No Child Left Behind, in terms of a decrease in physical activity and within our schools. Hopefully now we’ll start seeing that uptick with ESSA and with physical education, health education being considered a well-rounded subject.”
Characteristics of individuals		
Knowledge and self-efficacy	• State staff had greater knowledge and self-efficacy for 2 tools.	For somebody, like me, who has attended multiple trainings and oversees school health as an umbrella, I think it’s a little bit easier to grasp.
Delivery system		
Outer setting		
Student and family needs and resources	Parent support for school health	“Sometimes it’s the community or parent part of it. Cuz if you have parents that are really gung ho about making sure their kids are doin’ healthy lifestyles kind of stuff, then they can drive the administration.”
Inner setting		
Culture of wellness	A school’s health-related norms and values	“I think some of it is culture within the school, but also within the community.”
Relative priority	How nutrition and physical activity were prioritized in relation to academics and other concerns	“Whether it’s bullying or suicide prevention, or tobacco prevention, our guides around nutrition and physical activity are gonna be just one in a whole pool of guides.”
Readiness for implementation	• Leadership Engagement: Commitment and involvement of district and school leadership • Resources: A paid district wellness coordinator. • Access to knowledge and information. Teachers’ access to professional development related to the tools	“I see a principal as a gatekeeper and if that gatekeeper gets it, lots of these things are gonna be very impactful and effective.” “…being able to have something that works and put programs in place, and then following up to really see the results that come out of that. That’s through those health and wellness coordinators.” “If there isn’t a commitment to providing professional development, or dedicating dollars for training, or doing some sort of stipend for teachers, or covering for substitutes to get teachers trained… Then that policy’s not gonna go anywhere.”
Characteristics of individuals		
Knowledge, beliefs and self-efficacy	School staffs’ limited knowledge of CDC tools	“…your teachers and staff who don’t know about them [CDC tools], don’t know how to use them, or don’t know how to access them.”
Other personal attributes	School staffs’ motivation, particularly champions	“I think there’s a lot of intrinsic motivation that’s going on with the people who are champions within our region. I think it just takes a certain element of resiliency…”

#### Outer setting

Two factors in CFIR’s outer setting domain influenced support system staffs’ use of the tools: cosmopolitanism and external policy and incentives.

##### Cosmopolitanism (i.e., networked to other external organizations)

As noted above, state and district staff networked with numerous national, state, and local partners in their efforts to improve school health. For state staff, strong inter-relationships between those in state departments of health and education were particularly important. The partners shared innovative intervention strategies and implementation tools and assisted with trainings and technical assistance.

##### External policy and incentives

Federal policy and funding influenced state and district staff use of the tools. Staff frequently mentioned the shift from “No Child Left Behind” to the “Every Student Succeeds Act” and their hope that school districts would now have greater flexibility to invest resources in school health. State staff also noted that CDC funding requirements influenced which tools they used, particularly the priority given to environmental as opposed to curricular changes. CDC funding also strengthened relationships between staff in the departments of health and education, relationships that were identified as critical to promoting school health. These relationships were strengthened by the requirement that staff from the two departments create a memorandum of understanding and travel together to attend offsite training. Staff also noted a disconnect between CDC funding and the predominant sources of funding for school nutrition. District and school staff looked to the USDA, specifically the Team Nutrition initiative, for guidance and resources on school nutrition and were less likely to go to the CDC’s tools for guidance in this area.

State policy also influenced use of CDC tools, particularly state standards and regulations mandating health education, minimum weekly minutes of physical education and/or recess time, and school health boards. State staff also referenced the challenges created by local-level control over curricula.

#### Characteristics of individuals

Survey findings show that a majority of state staff were aware of three of the tools (85.5% - 91.3%) and reported very good or excellent levels of knowledge of and confidence to provide training on the CSPAP and the SHG (Table 4). They reported lower knowledge levels and training confidence for the HECAT and the P4HS. The tools for which staff had high levels of knowledge and confidence are also the tools for which they were more likely to have received training. When surveyed, 40% of state staff reported that they had received training on CSPAP, 23.5% on SHG, 7.5% on HECAT, and none had received training on the P4HS (Table 4).

**Table 4:** Individual-level data: CDC School Health Tools Online Survey, 2016

	CSPAP (*N* = 69)	HECAT (*N* = 69)	P4HS (*N* = 69)	SHG (*N* = 69)
Aware of tool	91.3%	85.5%	50.7%	89.9%
	CSPAP (*N* = 60)*	HECAT (*N* = 53)*	P4HS (*N* = 32)*	SHG (*N* = 51)*
Very good or excellent knowledge of tool	68.3%	26.4%	31.3%	54.9%
Confident or highly confident to train on tool	61.6%	26.4%	28.1%	60.8%
Have received training on tool	40.0%	7.5%	0.0%	23.5%

^a^Sample includes only those who were aware of the tool

*CSPAP* Comprehensive School Physical Activity Program, *HECAT* Health Education Curriculum Analysis Tool, *P4HS* Parents for Healthy Schools, *SHG* School Health Guidelines

### What contextual factors influenced delivery system staffs’ use of the CDC’s tools?

As summarized in Table 3, school staffs’ use of the tools was influenced by contextual factors within three CFIR domains: inner setting, outer setting, and characteristics of individuals.

#### Inner setting

Findings aligned with the following subset of constructs within the CFIR inner setting domain: culture of wellness, relative priority, and readiness for implementation.

##### Culture of wellness

District and local school staff frequently mentioned the importance of a culture of wellness to schools’ successful implementation of EBIs. The local culture sometimes worked against wellness such as in a school where a participant reported, “We pride ourselves on treats.”

##### Relative priority

Competing priorities were a particular concern for staff in local schools who were described as “super busy” and who had to include school health as one more item on a long list of priorities that included academic achievement, preventing violent and disruptive behaviors, and other health priorities (e.g., mental health and management of chronic conditions), among others.

##### Readiness for implementation

Findings aligned with the following CFIR readiness constructs: leadership engagement, available resources, and access to knowledge and information. Virtually, all participants identified *leadership engagement* as essential to the successful implementation of tools and EBIs. Leadership included superintendents, assistant superintendents, and principals. One of the most frequently identified *resources* was paid district wellness coordinators, who were present in three of the states that participated in interviews. In the fourth state, state staff identified the only district with a paid wellness coordinator as the most successful district in their state. *Access to knowledge and information* also contributed to implementation readiness. Teachers had limited access to professional development for both tools and EBIs as a result of limited funding for substitutes, travel, and registration fees.

#### Outer setting

Study findings supported one construct within the CFIR outer setting domain: *student and family needs and resources*. Staff in some but not all districts talked about the role that parents played in advocating for healthier school environments and practices.

#### Characteristics of individuals

Findings aligned with the CFIR constructs of knowledge and other personal attributes. In interviews, school staff evidenced limited knowledge of the tools. Participants frequently mentioned motivation as another personal attribute that influenced tool use, particularly the motivation of those serving as voluntary school wellness champions.

### What characteristics of the CDC’s tools influenced their use?

Delivery systems made only limited use of the tools, primarily because of their high levels of complexity. We therefore report on the characteristics of tools that influenced their use by support systems staff (see Table 5). Seven of the characteristics align with those characteristics identified in the CFIR. The eighth, “compatibility,” was inductively derived during coding (note: CFIR categorizes compatibility in the inner setting domain) [14].

**Table 5:** Characteristics of CDC tools that influence support system use [14]

Characteristics of tools		
Credibility of source	Value of CDC as the source of the tools	“I really appreciate the tools that come out of CDC with them being research-based and best practice. Because with prevention, that’s really all we have behind us is to tell people that it works.”
Evidence strength and quality	• The tools’ guidance is supported by evidence	“SHG it’s always, for me, been a go-to document. It’s very rich. Rich with the evidence and the strategies”
Compatibility	• Congruence with state, district, and school priorities, resources, and needs and with tools already using	“In terms of the education component, we never had much of a role in supporting health education. It was always around physical education, because clearly it’s the one area that supports not only physical activity, but physical education and the components of physical education. Also the link to cognitive improvement and academic achievement through physical activity and movement. That’s been our primary focus”
Complexity	• Tools are long and complicated • State- and district-level staff appreciated tools’ comprehensiveness • Major barrier to school staffs’ use of tools	“It depends on who your end user is, I guess. For somebody, like me, who has attended multiple trainings and oversees school health as an umbrella, I think it’s a little bit easier to grasp. For some of my school people, who are really just looking for the biggest bang for their buck, “What can I do?”, boots on the ground, I think it’s a little bit less user-friendly.”
Relative advantage	Benefit (or lack of) that CDC tools provided relative to other tools	SPARK book is described as more useable than CDC tool because it “is set up to where you can basically just read it off and have instant activities. You don’t even have to see the lesson. You just look at it be like ‘This is what we’re doing.’ Boom…[SPARK is] super ready to go. You just take one page and one page, put ‘em together.”
Adaptability	Value of being able to extract and use one or more components of a tool	I really tried to educate our local health departments to go out into the schools and have them do the assessments of the components, and then approach and say it looks like you’re doing three out of the five components of this framework of CSPAP. How can we incorporate or get another component, work on a component for this year?
Design quality and packaging	Perceptions of how well the tool is assembled and presented	“the user friendly of how it looks, how it can be pieced together, how it can be paired together, [and] the sections.”

#### Credibility of source and evidence strength and quality

State and district staff strongly endorsed the credibility of the CDC as the source of tools. They viewed the CDC as the best source of strong, high quality evidence to make the case for *why* to implement EBIs and also for *what EBIs* will improve school environments, policies, and curricula.

#### Compatibility

Participants most frequently described compatibility as the factor differentiating which of the four tools they used most. Their reasons for using the CSPAP and SHG more than the other two were largely related to how well those tools aligned with their priorities, existing resources and needs, and with the tools they were already using. State staff reported that the CSPAP and SHG aligned with the goals of CDC’s school health funding. Conversely, state staff reported that they did not promote or support HECAT, in part, because health education curricula are not a priority for the CDC funding and, in most states, are under the control of individual school districts. District staff did not use HECAT because they lacked the time required to develop their own curriculum or to do an in-depth review of existing curricula. Neither state- nor district staff used the P4HS, in part, because they could not identify an appropriate audience for the tool since teachers primarily communicate with parents, and do so through in-person meetings.

#### Complexity, relative advantage, and adaptability

State and district staff reported that the HECAT, CSPAP, and SHG were “daunting,” “a beast,” and “really long” and that their *complexity* made them challenging to use. They were willing to manage a tool’s complexity, however, in exchange for the comprehensive and integrated overview a tool provided, which offered a *relative advantage* in comparison to other tools. The SHG’s and CSPAP’s comprehensive menu of EBIs were viewed as having advantages relative to other tools to improve school health.

State and district staff reported that the challenges of complexity could be overcome when the tools were adaptable, with *adaptability* described as the ability to extract and use one or more components of a tool, rather than having to use it all. For example, district staff talked about providing schools with the CSPAP’s templates for action planning, the SHG’s executive summary, and the P4HS’s one-page “Ideas for Parents” for schools to include in their newsletters.

#### Design quality and packaging

Participants were most likely to identify positive aspects of the CSPAP’s and P4HS’s design quality and packaging and to note that the SHGs and HECAT were too dense. When asked how the CDC’s tools could be improved, participants often made suggestions related to design quality and packaging. For example, they recommended creating (1) online, interactive formats, (2) condensed or simplified versions or executive summaries, (3) and ready-to-use materials like presentation PowerPoints.

## Discussion

Use of school health implementation tools occurs within a multi-tiered, “interactive system” [13], with those working in local schools (i.e., the delivery system) making limited use of the tools to implement EBIs, and those working in school districts and state departments of health and education (i.e., support systems) integrating the tools with the promotion, training, technical assistance, and other strategies they used to support schools’ EBI implementation. Previous studies have identified the importance of training, technical assistance, and other support strategies to the success of school health [20–23]. In one of the few studies to assess the association between support strategies and implementation, Hager et al. [24] found that schools whose staff reported high levels of school district support were more likely to implement local wellness policies. Finally, of particular, relevance to this study, Cradock et al. [25] described the strategies that state staff used to promote and support physical education and activity policies. The strategies they identified were similar to this study’s findings and included communication, professional development, and technical assistance.

This study found that most of those working in the support system were using two of the four CDC tools studied—the CSPAP and SHG—and fewer were using the HECAT and P4HS. Several reasons may explain underuse of the HECAT and P4HS. First, the state staff were less likely to have been trained on the HECAT and P4HS. Second, P4HS was the tool most recently released (2015). Notably, in a systematic review of 26 comprehensive school health interventions, Langford et al. [21] found that engaging families was the most challenging and least successful component of the interventions reviewed. The difficulty of schools engaging families also was a key finding of Agron et al.’s [26] national study of factors influencing the implementation of school wellness policies and may be another factor contributing to the limited uptake of P4HS. As summarized below, variations in the use of the tools also were influenced by contextual factors and by characteristics of the tools.

### Findings related to contextual factors that influenced use of CDC tools

In the outer setting domain, support system staffs’ use of tools was positively influenced by staff engagement with other organizations involved in school health (i.e., cosmopolitanism) and state and federal policy that were supportive of tools’ objectives. Within the inner setting domain, factors that influenced delivery system staffs’ use of the tools included a culture of wellness, prioritization of school health, and readiness to implement EBIs, with readiness characterized by the presence of engaged leadership, a wellness coordinator, and support for professional development. In the characteristics of individual domain, both support and delivery system staff’s knowledge and self-efficacy influenced use of the tools.

Previous studies of contextual factors influencing schools’ implementation of school health interventions have identified factors similar to those identified in this study. Identified factors include those in CFIR’s individual and inner setting domains, such as knowledgeable and motivated staff, engaged leadership, a dedicated school champion or wellness coordinator, and the relative priority given to school health [20–24]. Our study is distinct in that it is one of the first to identify factors that influenced support systems’ use of tools, including multiple factors in CFIR’s outer setting domain.

### Characteristics of CDC tools that influenced their use

Across CDC tools, support system staff valued the “credibility of the source” (CDC) and the tools’ “evidence strength and quality” and viewed the tools’ “complexity” as a barrier. Participants were willing to manage a tools’ complexity if it was viewed as having a relative advantage compared to other tools and if it had high levels of adaptability. A tool’s compatibility with a support system’s needs was the primary factor explaining why some tools were used more than others.

The relative value of the different characteristics varied depending on participants’ purpose for using the tool. Support system staff used tools for three purposes—to make the case for *why* EBIs are important, to identify *what* EBIs to adopt, and to provide guidance on *how to* implement EBIs in schools. When using tools to make the case for *why* to use EBIs and *what* EBIs to adopt, support system staff highly valued the CDC tools’ “credibility of source” and “evidence strength and quality.” They were willing to accommodate the tools’ complexity in exchange for comprehensiveness and a strong evidence base. The tools’ “complexity” was a barrier to staff in local schools, who did not have the time needed to search for and extract *how to* guidance. Because of this, state and district staff appreciated tools with high levels of “adaptability,” which allowed them to extract components of the tools and format them for use by those working in local schools.

Previous studies have addressed the importance of implementation tools and the characteristics of both interventions and tools that influence their use, most notably the importance of adaptability. In a review of six qualitative studies, Hung et al. [22] identified the availability of frameworks and guidelines as one of five key enablers, and Agron et al. [26] identified the absence of tools as one of the top four barriers to the implementation of school health interventions. Hung et al. further noted the value of frameworks and guidelines that structure implementation into phases with specific aims and milestones and the importance of being able to adapt them to specific contexts. Similarly, in a literature review of 26 studies, Langford et al. [21] found that comprehensive approaches to improving school health were most likely to be successful if they could be adapted to schools’ needs, resources, and communities.

### Implications of study findings for the design of implementation tools

Study findings suggest that formative work with an implementation tools’ intended users is essential to designing tools that meet those users’ needs and align with their practice context. In this study, we found that implementation tools need to be designed differently for those working in support systems versus those working in delivery systems. For those working in support systems, complex, in-depth tools may be appropriate if they provide a comprehensive, integrated overview of EBIs for a multipronged, evidence-supported approach to improving school health. Delivery systems, on the other hand, need ready-to-use tools. Rather than trying to make tools that work for both support and delivery systems, synthesis and translation systems (e.g., the CDC) may want to invest in developing tools for support systems and collaborate with organizations that may be more closely connected to delivery systems and, therefore, better suited to creating tools for delivery systems to use. Synthesis and translation systems also might train support providers on appropriate ways to extract and adapt components of their tools so that they meet the “how to” needs of delivery systems.

Study findings also suggest that tools need to be designed to align with the inner and outer settings of both support and delivery systems and with characteristics of the individuals who will be using the tools. Formative work with the tools’ intended users can aid in identifying their goals for the tools and the characteristics of tools they view as most important. Formative work also can be used to identify contextual factors that may influence tool use. In addition to guiding tool design, the identified contextual factors may be translated into assessment tools that support providers might use to identify and address gaps in capacity prior to attempting to implement EBIs (e.g., lack of knowledge or leadership engagement).

Study findings included preferred approaches to technical assistance, which also have implications for the design of implementation tools. Specifically, tools may be most useful if they are designed to be easily adapted and include tools for assessing performance and guidance for setting achievable goals in response to assessment findings.

### Implications of study findings to the application of CFIR to implementation tools

This study demonstrates the applicability of four of the CFIR’s five domains to the study of contextual factors influencing the use of implementation tools (characteristics of the intervention [i.e., tool], inner setting, outer setting, and individuals). Findings suggest that CFIR may be a useful framework for use in formative work to identify factors that need to be considered so that tools are designed to align with user’s needs, preference, and contexts. We replaced the fifth CFIR domain, process, with the ISF’s concept of innovation support strategies. The CFIR process domain captures the concept of “support systems” though inclusion of “external change agents” as one of its constructs. The CFIR does not, however, describe the processes that external change agents use to influence implementation. To address this gap, we drew on the ISF and other frameworks to describe support system strategies to include promotion, training, and technical assistance.

During coding, “compatibility” emerged as a characteristic that influenced tool use and this may suggest a revision to the CFIR framework, which locates “compatibility” within the inner sitting domain. Our findings fit with Diffusion of Innovations (DOI) theory, which identifies compatibility as one of five innovation attributes that influence the decision to adopt [27]. Of note, DOI theory defines compatibility in relation to how an innovation “is perceived” whereas the CFIR defines in terms of “tangible fit.” [14] The fact that this study’s findings were based solely on perceptions may explain why compatibility fell within the characteristics of the tool domain. In talking about compatibility, participants described the contextual factors that determined the tool’s compatibility, but generally described compatibility as a feature of the tool rather than the context.

### Limitations and need for further research

This study’s survey response rate was only 38.8%, and therefore, survey findings should be interpreted with caution. For the site visits to school districts, we purposefully selected districts that were recognized leaders in school health implementation, and therefore findings, may not be generalizable to all school districts. Findings may not translate to school districts with even more competing demands and lower levels of capacity to use school health tools, which may be the school districts with students at greatest risk for poor health outcomes.

Survey data collected in this study are self-reported and cross sectional. Further research is needed to test the effectiveness of school implementation tools together with other implementations strategies over time. This might include studies that compare the effects of training and technical assistance with and without a tool, or directly compare the effectiveness of two different types of tools. These studies may assess tools’ effectiveness at improving school health environments, policies, and practices, and also students’ health behaviors. To further identify which tools have the greatest potential to impact health, these studies might assess the number and representativeness of schools and school districts that adopt the tools, the cost, and feasibility of tool use and whether tools are maintained over time.

## Conclusions

This study illustrates how CFIR and the ISF might be applied to evaluate factors influencing the use of implementation tools in both support and delivery systems. These factors may be applied to improve the design of school health and potentially other types of implementation tools that are applied across multi-tiered support and delivery systems, such as the American Cancer Society and other organizations, that are developing implementation tools for use by practice facilitators and other exchange agents working with community clinics and other delivery settings.
